# Application of Berberine on Treating Type 2 Diabetes Mellitus

**DOI:** 10.1155/2015/905749

**Published:** 2015-03-11

**Authors:** Bing Pang, Lin-Hua Zhao, Qiang Zhou, Tian-Yu Zhao, Han Wang, Cheng-Juan Gu, Xiao-Lin Tong

**Affiliations:** ^1^Department of Endocrinology, Guang'anmen Hospital of China, Academy of Chinese Medical Sciences, Beijing 100054, China; ^2^Laboratory of Molecular and Biology, Guang'anmen Hospital of China Academy of Chinese Medical Sciences, Beijing 100054, China; ^3^Department of Digestion, Beijing Hospital of Traditional Chinese Medicine, Capital University of Medicine Sciences, Beijing 100010, China

## Abstract

Traditional Chinese medicine (TCM) performs a good clinical practice and is showing a bright future in the treatment of diabetes mellitus (DM). TCM treatment has certain advantages of less toxicity and/or side effects, and herbs could provide multiple therapeutic effects. Berberine (BBR) is a classical natural medicine. In this review, we summarize the application of BBR in the treatment of DM from two aspects. First, modern pharmacological effects of BBR on glucose metabolism are summarized, such as improving insulin resistance, promoting insulin secretion, inhibiting gluconeogenesis in liver, stimulating glycolysis in peripheral tissue cells, modulating gut microbiota, reducing intestinal absorption of glucose, and regulating lipid metabolism. BBR is used to treat diabetic nephropathy (DPN), diabetic neuropathy (DN), and diabetic cardiomyopathy due to its antioxidant and anti-inflammatory activities. Second, the clinical application of BBR is reviewed, such as listing some clinical trials on the effectiveness and safety of BBR, explaining applicable stage and syndrome, the reasonable dose and dose formulation, and the toxicity and/or side effects. This review provides scientific evidence about BBR, as well as introducing some traditional Chinese medical theory and clinical experience, in order to guide clinician to use BBR more suitably and reasonably.

## 1. Introduction

Diabetes mellitus (DM) is a metabolic disorder in the endocrine system. According to the statistics from the International Diabetes Federation (IDF), the global prevalence of DM among adults aged 20–79 years was 8.3% in 2013, and the total number of patients was estimated to be 382 million globally, which was predicted to increase to nearly 592 million by 2035 [1]. The high incidence of DM has a significant impact on the quality of life as well as on the economic cost of the health care system, which represents a major public health issue [2]. Type 2 diabetes mellitus (T2DM) is the predominant form of DM that accounts for 90–95% of diabetic patients globally [3, 4]. The standard therapy for T2DM includes balanced diet, appropriate exercise, use of oral hypoglycemic drugs, and/or subcutaneous insulin injections [5]. Although considerable progress has been made in the field of antidiabetic drugs including oral hypoglycemic agents (OHA) and insulin, some shortages still exist. The efficacy of the antidiabetic drugs in achieving optimal glycemic control was only 41%, which was far from satisfactory [6, 7], and no drug could maintain stable blood glucose control for years [8]. In addition, more and more patients have been concerned about their potential toxicity and side effects of antidiabetic drugs, such as weight gain, bone loss, and increased risk of cardiovascular events [8, 9]. For example, the potential adverse effects of sulfonylureas include hypoglycemia, weight gain, and cardiovascular damage [10]; main concern about metformin is gastrointestinal discomfort; the use of pioglitazone has been thought to increase risks of bladder cancer, edema, distal bone fractures in postmenopausal women, and so forth [8]. These chemical or biochemical agents also have their contraindications; for example, metformin is limited in diabetic patients with renal impairment, hepatic disease, or cardiopulmonary insufficiency [11]. Natural medicines, especially Chinese herbal medicines, have been demonstrated to possess a mild but significant antihyperglycemic effect [12, 13] and the long-term use of these herbal medicines may be helpful in alleviating some diabetic complications [2, 3, 13]. Additionally, the use of herbal medicine in combination with Western drugs permits lower doses of the drug and/or decreases frequency of administration which reduces the adverse effects, as well as improving better efficacies [14]. Unlike Western medicine which usually contains a single active ingredient aiming for a specific mechanism, Chinese herbal medicines, which include various ingredients, have advantages of holistic regulation, which may regulate the functions of body through multiple targets and multiple mechanisms, therefore obtaining satisfactory efficacies in alleviating metabolic disorders (obesity, hypertension, and dyslipidemia) and improving the diabetic symptoms and quality of life. In addition, herbal medicines possess less toxicity and/or side effects and are less costly than Western medicine. Thus, herbal medicine could be thought of as a good alternative or at least supplement to Western hypoglycemic agents to treat DM [13, 15, 16]. There are 86 herbal medicines often used in the traditional Chinese formulas for T2DM and its complications [13]. Among these, berberine (BBR) has aroused interests in its great antihyperglycemic and hypolipidemic activities [17, 18]. Moreover, BBR has shown positive effect in treating diabetic nephropathy (DPN), diabetic neuropathy (DN), and diabetic cardiomyopathy. In this review, we systematically summarize the scientific evidence about BBR's antidiabetic mechanisms, its clinical effectiveness, and safety in the treatment of T2DM, in order to guide clinicians to use BBR more suitably and reasonably in the clinical practice. The literature is acquired from both English and Chinese electronic databases, including MEDLINE (1959–2014), PUBMED (1959–2014), EMBASE, Chinese Biomedical Literature Database (CBM) (1978–2014), Chinese National Knowledge Infrastructure (CNKI) (1980–2014), Chinese Scientific Journal Database (VIP) (1989–2014), and WANFANG Databases (1998–2014).

## 2. Researches on Berberine

### 2.1. Chemical Characteristics and Source of Berberine

**Figure 1:**
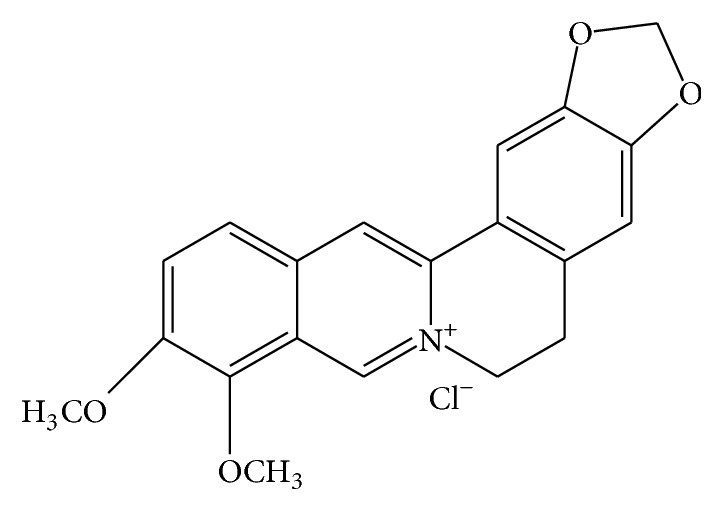
Chemical structure of BBR.

BBR is a plant quaternary ammonium salt from the group of isoquinoline alkaloid (2,3-methylenedioxy-9,10-dimethoxyprotoberberine chloride; C_20_H_18_NO_4_^+^) (Figure 1), with a molar mass of 336.36122 g/mol [19]. It can be isolated from a variety of plants, such as* Coptis chinensis* (Coptis or Goldthread),* Hydrastis canadensis* (goldenseal),* Berberis aquifolium* (Oregon grape),* Berberis aristata* (Tree Turmeric)* and Berberis vulgaris* (Barberry), and* Arcangelisia flava* [20].

### 2.2. Researches on Antidiabetic Mechanism of Berberine

**Figure 2:**
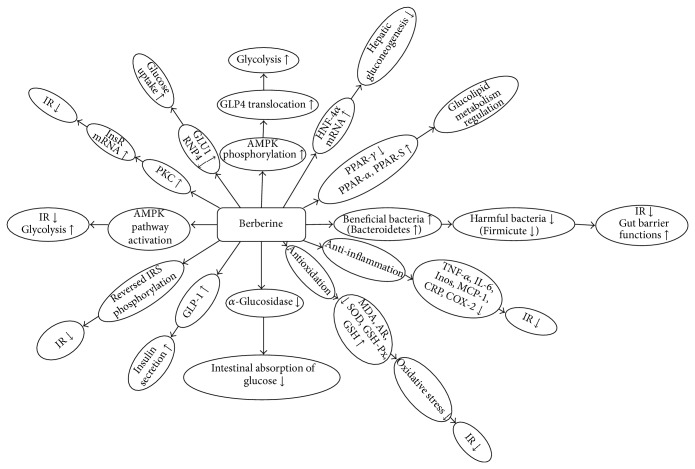
Main mechanisms of BBR on glucose metabolism. Note: AMPK: adenosine monophosphate- (AMP-) activated protein kinase; HNF-4: hepatic nuclear factor 4 alpha; PPARs: peroxisome proliferator-activated receptors; TNF-*α*: tumor necrosis factor-*α*; IL-6: interleukin-6; INOS: inducible nitric oxide synthase; MCP-1: monocyte chemoattractant protein-1; CRP: C-reaction protein; COX-2: cyclooxygenase-2; IR: insulin resistance; MDA: malondialdehyde; AR: aldose reductase; SOD: superoxide dismutase; GSH-PX: glutathione peroxidase; GSH: glutathione; GLP-1: glucagon-like protein-1; PKC: protein kinase C; InsR: insulin receptor; GLU1: glucose transporter type 1; RNP4: retinol binding protein 4; GLUT4: glucose transporter type 4.

**Figure 3:**
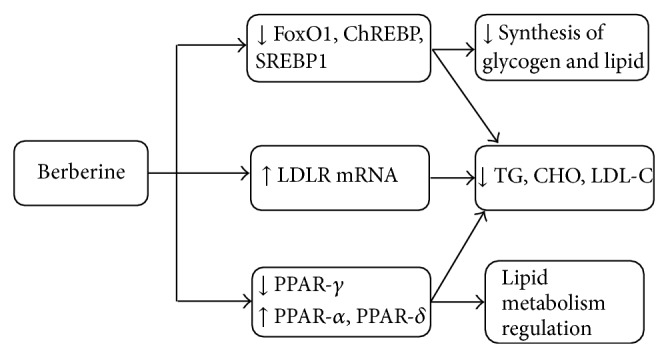
Main mechanisms of BBR on lipid metabolism. Note: FoxO1: Forkhead transcription factor O1; ChREBP: carbohydrate responsive element-binding protein; SREBP1: sterol regulatory element-binding protein-1; LDLR mRNA: low-density lipoprotein receptor m ribonucleic acid; TG: triglyceride; CHO: cholesterol; LDL-C: low-density lipoprotein cholesterol; PPARs: peroxisome proliferator-activated receptors.

BBR has been used in traditional Chinese medicine and Ayurvedic medicine for its antimicrobial activity, antiprotozoal activity, and antidiarrheal activity to treat bacterial diarrhea [20]. Many studies have indicated that BBR possesses multispectrum therapeutic activities; it may be effective in treating diverse chronic diseases, including diabetes (antihyperglycemia), cardiovascular diseases (antihyperlipidemia and antihypertension), cancer (the cytotoxic effect, the inhibitory effects on the proliferation and reproduction of certain tumorigenic microorganisms and viruses), depression, and inflammatory diseases (anti-inflammatory activity) [21]. The antihyperglycemic activities of BBR were firstly observed in 1986 [22]. BBR regulates glucose metabolism possibly through multiple mechanisms and signal pathways, such as increasing insulin sensitivity, activating the adenosine monophosphate- (AMP-) activated protein kinase (AMPK) pathway, modulating gut microbiota, inhibiting gluconeogenesis in liver, stimulating glycolysis in peripheral tissue cells, promoting intestinal glucagon-like protein-1 (GLP-1) secretion, upregulating hepatic low-density lipoprotein receptor mRNA expression, and increasing glucose transporter [19–21, 23]. Main mechanisms of BBR on glucose and lipid metabolism are shown in Figures 2 and 3.

#### 2.2.1. Effects of Berberine on Insulin Sensitivity and Insulin Secretion

Protective effects of BBR on islet function may involve two pathways. Firstly, BBR improved insulin sensitivity in T2DM characterized by insulin resistance [24]. Moreover, BBR promoted insulin secretion and protected pancreatic islet cell via antioxidant activity in type 1 diabetes mellitus (T1DM) and late stage of T2DM characterized by damaged islet function [25, 26]. Kong et al. [27] investigated the molecular mechanism of BBR against insulin resistance, and BBR was demonstrated to lower the fasting blood glucose (FBG) and fasting serum insulin via upregulating insulin receptor (InsR) expression both in vitro and in animal experiments. Subsequently, they investigated BBR increased InsR messenger RNA (mRNA) and protein expression through protein kinase C- (PKC-) dependent activation of its promoter in a variety of human cell lines. In adipose and muscle cell, several researches demonstrated that BBR may stimulate glucose uptake into cell by upregulating glucose transporter type 1 (GLUT1) expression [28] and inhibiting retinol binding protein-4 (RBP-4), which can act as an effective insulin sensitizing function [29, 30]. It also has effects on phosphorylation of InsR and insulin receptor substrate-1 (IRS-1) [31], finally resulting in alleviating insulin resistance. Lee et al. [32] demonstrated that BBR upregulated adenosine monophosphate- (AMP-) activated protein kinase (AMPK), which results in improvement of insulin resistance and promotion of glycolysis. AMPK is a major energy-sensing/signaling protein kinase, which belongs to metabolite-sensing protein kinase family that acts as a fuel gauge by monitoring cellular energy levels, thus preventing metabolic disorders [33, 34]. Inhibition of mitochondrial oxidation by BBR and increased AMP/ATP ratio causes AMPK activation [35]. BBR significantly promoted the phosphorylation of AMPK*α* subunit, which seems to be another important way to activate AMPK [28, 36]. In in vitro experiment, AMPK phosphorylation was increased by BBR at 0.5 h, and the increase remained for ≥16 h [35]. On AMPK activation, BBR increases glucose transport activity of 3T3-L1 adipocytes as well as glucose transporter type 4 (GLUT4) translocation in L6 cells; then the glycolysis increased and the level of blood glucose decreased [28].

BBR may significantly promote GLP-1 secretion, thus promoting insulin secretion and improving the function of *β*-cells in the pancreas [37, 38]. GLP-1 receptors played important roles in islet cell survival. When GLP-1 receptors were activated, adenylyl cyclase was simultaneously activated and cyclic AMP (cAMP) was generated, and the second messenger pathways were open and adenosine triphosphate- (ATP-) dependent K^+^ channels closed. Calcium influx through the voltage-dependent calcium channels happened due to increased intracellular potassium. The increase of intracellular Ca^2+^ promoted the migration and exocytosis of the insulin granules [19]. BBR also was reported to increase glucose-stimulated insulin secretion and proliferation in Min6 cells via activation of insulin/insulin-like growth factor-1 signaling cascade [29].

#### 2.2.2. Effects of Berberine on Glucolipid Metabolism in Liver

Liver plays an important role in glucolipid metabolism, in which BBR exerts main effects to regulate the glucolipid metabolism via intranuclear transcription factors regulation. There are several transcription factors that participated in glycogen and lipid synthesis, including Forkhead transcription factor O1 (FoxO1), sterol regulatory element-binding protein 1c (SREBP1), and carbohydrate responsive element-binding protein (ChREBP). Xia et al. [39] demonstrated that expression of these three transcription factors could be reduced by BBR. Furthermore, BBR lowered blood glucose by inhibiting hepatic gluconeogenesis directly. Hepatic nuclear factor 4 alpha (HNF-4*α*) is a factor from hepatic nucleus family, which could inhibit gluconeogenesis. Researches demonstrated that BBR may inhibit hepatic gluconeogenesis and lower the blood glucose by increasing HNF-4*α* mRNA expression and hepatic steatosis, and expression of fatty acid synthase (FAS) was also reduced [40]. Other researches showed that BBR improved metabolic activities possibly through modulating the peroxisome proliferator-activated receptors (PPARs) protein expression in liver. PPARs are ligand dependent transcription factors, which are composed of PPAR-*α*, PPAR-*δ*, and PPAR-*γ*, and play important roles in regulating target genes expression with regard to glucolipid metabolism [41]. Zhou et al. [42] showed that BBR alleviated the pathological progression of liver, reducing the hepatic glycogen, modulating glucolipid metabolism possibly through increasing PPAR-*α* and PPAR-*δ* proteins expression, which contributed to improving of lipid metabolism, and significantly reducing PPAR-*γ* expression in liver. The PPAR-*γ* agonists may have therapeutic effects on metabolic disorders by increasing fatty acid consumption in skeletal muscle and adipose tissue [41, 43]. Huang et al. [44] demonstrated that BBR inhibited PPAR-*γ* mRNA and protein levels in 3T3-L1 preadipocytes, and BBR inhibited the full-length PPAR-*γ* and PPAR-*α* transcription activity by reporter gene assays.

#### 2.2.3. Effects of Berberine on Reducing Intestinal Absorption of Glucose

BBR may reduce intestinal glucose absorption to lower the blood glucose, which is achieved by inhibiting *α*-glucosidase activity. *α*-Glucosidase is an intestinal enzyme for digesting carbohydrates and converting into monosaccharides; the inhibition of this enzyme makes digestion and absorption of carbohydrates be suppressed [45]. BBR could suppress the expression of intestinal disaccharidases in diabetic rat and Caco-2 cells, with the most significant effect found in the duodenum [46]. Liu et al. [47] also reported that BBR downregulates the *β*-glucuronidase in diabetic rats, thus improving the glycol tolerance of streptozotocin-induced diabetic rats, which is similar to acarbose.

#### 2.2.4. Effect of Berberine on Modulating Gut Microbiota

BBR possesses significant antimicrobial activity, which may be related to its antidiabetic mechanism. Han et al. [48] proposed a hypothesis that BBR was used to treat T2DM through modulating gut microbiota. The occurrence of T2DM is related to changes in gut microbiota composition, which caused activation of a network of inflammatory signal pathways via the lipopolysaccharides (LPS) and CD14/toll-like receptor 4- (TLR4-) dependent pathway, and then made the body in a state of low-grade inflammation; ultimately, T2DM came into being [49, 50]. Probiotic and antibiotic agents have been shown to play important roles in the treatment of T2DM. BBR has significant antimicrobial activity against bacterial, viral, and fungal infections as well as parasites and worms [20, 51–53]. Therefore, BBR may exert antidiabetes effects via regulating gut microbiota. Bacteroidetes and Firmicutes are two types of gut microbiota that affect energy metabolism homeostasis, and some studies suggested that obese humans or animals have more Firmicutes and less Bacteroidetes than lean couples controls [54, 55]. Xie et al. [56] investigated the effects of BBR on gut microbes in high-fat diet-fed (HFD) mice. Results showed that BBR significantly lowered the levels of blood glucose and lipids; moreover, BBR significantly reduced the number of Firmicutes and increased that of Bacteroidetes in the feces of HFD-fed mice. In Zhang's research [57], the prevention of obesity and insulin resistance by BBR in HFD-fed rats is possibly regulated by structural modulation of the gut microbiota, which may help to alleviate inflammation by reducing the exogenous antigen load in the host and elevating short-chain fatty acid (SCFA) levels in the intestine. BBR was shown to enrich SCFA-producing bacteria; the beneficial effects of SCFAs included improving gut barrier functions, alleviating inflammation, or creating a nonpermissive environment for pathogens, which may also help to improve obesity and insulin resistance-related metabolic abnormalities.

#### 2.2.5. Antioxidant and Anti-Inflammatory Activities of Berberine

Oxidative stress and inflammation are associated with the pathogenesis of DM and its complications [58, 59]. Recent studies showed that BBR could also regulate glucose homeostasis through decreasing oxidative stress by changing oxidative stress markers, antioxidant enzymes, and proinflammatory cytokines [58, 60, 61]. BBR administration could reduce malondialdehyde (MDA) content and increase the contents of superoxide dismutase (SOD), glutathione peroxidase (GSH-Px), and glutathione (GSH) in diabetic animals, which help to seek for excessive free radicals and protect oxidative stress [62, 63]. However, in one study this conclusion appeared to be controversial. Wang et al. [64] indicated that the levels of MDA and SOD of the diabetic animals did not have significant difference statistically compared with those of the normal control animals (*P* > 0.05), suggesting that the effect of BBR on oxidative stress was not obvious. Therefore, further researches are needed to explore this mechanism. BBR has a good therapeutic efficacy for DN. Liu et al. [65] found that BBR ameliorated renal injury in streptozotocin-induced Wistar rats by inhibiting aldose reductase and oxidative stress. After the treatment with oral administration of BBR (200 mg·kg^−1^·d^−1^), FBG, blood urea nitrogen (BUN), serum creatinine (Cr), and 24 h urinary albumin (24 h-UAlb) were significantly decreased, and serum SOD activity was increased, while the content of MDA, aldose reductase (AR) activity, and the expression of AR mRNA and protein in the kidney were markedly decreased compared with control group (*P* < 0.05). Lan et al. showed that the ratio of kidney weight to body weight decreased, and synthesis of TGF-*β*1 was suppressed after BBR treatment [66]. The antioxidant effect was also observed in rat mesangial cells cultured with high glucose-containing media [67], and the mechanisms that BBR ameliorated renal injury possibly were related to suppression of sphk-s1p signaling pathway [66] as well as aldose reductase (AR) activity in vitro [68].

In current medicine, it is generally accepted that DM is usually associated with chronic subclinical inflammation, and the roles of inflammation in the pathogenesis of T2DM and its vascular complications have been confirmed by several researches [59, 69]. The anti-inflammatory effects of BBR have been observed in vitro and in vivo, which demonstrated that BBR exerted the hypoglycemic effects possibly via controlling inflammation [59]. BBR treatment could inhibit the expression of proinflammatory cytokines as well as acute phase proteins, such as tumor necrosis factor-*α* (TNF-*α*), interleukin-1*β* (IL-1*β*), interleukin-6 (IL-6), prostaglandins (PGs), inducible nitric oxide synthase (iNOS), matrix metalloprotease 9 (MMP9), monocyte chemoattractant protein-1 (MCP-1), C-reaction protein (CRP), and cyclooxygenase-2 (COX-2) [70]. In a research, the insulin sensitizing effect of BBR was also associated with its anti-inflammatory activity. Lou et al. showed that BBR significantly increased insulin-mediated tyrosine phosphorylation of insulin receptor substrate-1 (IRS-1) in HepG2 cells with a decrease of cytokine production and serine phosphorylation [71]. BBR suppresses inflammation through various mechanisms, such as the AMPK, mitogen-activated protein kinase- (MAPK-) mediated pathways, the nuclear factor-kappa B (NF-*κ*B), and the Rho GTPase signaling pathway [58, 70, 72, 73].

#### 2.2.6. Effects of Berberine on Lipid Metabolism

BBR showed antihyperlipidemia effect on lowering the levels of total cholesterol (CHO), triglyceride (TG), and low-density lipoprotein cholesterol (LDL-C), which has been observed in both animals and humans [74]. Kong et al. [75–77] found that BBR upregulated the expression of hepatic low-density lipoprotein receptor (LDLR), mainly dependent on stabilizing LDLR message ribonucleic acid mRNA in an extracellular signal-regulated kinase (ERK) pathway. Lee et al. [78] demonstrated that BBR induced LDLR expression mainly dependent on activating c-Jun N-terminal kinase (JNK) way. Brusq et al. [79] showed that BBR also activates AMPK, resulting in inhibition of CHO and TG synthesis in human hepatocytes, and these effects could explain the strong reduction of plasma TGs observed with BBR in clinical trials.

## 3. Clinical Effectiveness and Safety of BBR

### 3.1. Some Clinical Trials Observing the Effectiveness of BBR

#### 3.1.1. Treatment Group with BBR Alone

The glucose-lowering activity of* RC* has been confirmed by several clinical trials. Yin et al. [80] investigated the efficacy of BBR in a pilot study; 36 adults with newly diagnosed T2DM were randomly assigned to BBR or metformin treatment (500 mg three times a day) in the first 3-month study. Results showed that BBR significantly lowered hemoglobin A1c (HbA1C) (from 9.5 ± 0.5 to 7.5 ± 0.4%, *P* < 0.01), FBG (from 10.6 ± 0.9 to 6.9 ± 0.5 mmol/L, *P* < 0.01), postload plasma glucose (PBG) (from 19.8 ± 1.7 to 11.1 ± 0.9 mmol/L, *P* < 0.01), and TG (from 1.13 ± 0.13 to 0.89 ± 0.03 mmol/L, *P* < 0.05) in patients with T2DM. In a second study, 48 adults with poorly controlled T2DM were treated with BBR supplementary for 3 months. There was a significant decrease in HbA1c (from 8.1 ± 0.2 to 7.3 ± 0.3%, *P* < 0.001), FBG, PBG, CHO, and LDL-C. Fasting plasma insulin and HOMA-IR were reduced by 28.1% and 44.7% (*P* < 0.001), respectively. The hypoglycemic effect of BBR was similar to that of metformin, and no significant changes of alanine aminotransferase (ALT), *γ*-glutamate transpeptidase (*γ*-GT), and Cr were observed in the treated group. This study indicated that BBR was a potent oral hypoglycemic agent that also had beneficial effects on lipid metabolism. Zhang et al. [81] showed that BBR (1 g/d, for 2 months) significantly lowered FBG by 25.9% (*P* < 0.001), HbA1c by 18.1% (*P* < 0.001), TG by 17.6% (*P* < 0.01), and insulin levels in ninety-seven patients with T2DM. The FBG- and hemoglobin A1c-lowering efficacies of BBR were close to those of metformin (1.5 g/d) and rosiglitazone (4 mg/d), and the percentages of peripheral blood lymphocytes which express InsR were increased after treating with BBR, suggesting that the glucose-lowering efficacy of BBR has relationship with its activities on InsR in humans. TG-lowering efficacy of BBR was better than those of metformin and rosiglitazone, and no adverse effects were showed in the BBR-treated patients. Zhang et al. [82] enrolled 116 patients diagnosed with T2DM and dyslipidemia. The patients were randomly divided into two groups, receiving 1 g/day of BBR or placebo for 3 months. After 3 months of treatment, results showed significant reductions in BBR group for HbA1c (from 7.5 ± 1.0 to 6.6 ± 0.7%, *P* < 0.0001), FBG (from 7.0 ± 0.8 to 5.6 ± 0.9 mmol/L, *P* < 0.0001), and PBG (from 12.0 ± 2.7 to 8.9 ± 2.8 mmol/L, *P* < 0.0001). There was also a significant decrease in CHO, TG, and LDL-C. Moreover, the glucose disposal rate was increased after treatment with BBR (*P* = 0.037), but there was no significant change found between two groups.

#### 3.1.2. Treatment Group with BBR Plus Western Medicine

In many trials, BBR was used in combination with Western medicines, such as glibenclamide, metformin, or rosiglitazone, and indicated that they had better efficacies in lowering blood glucose and improving diabetic symptoms than Western medicine alone in T2DM patients. In a study conducted by Sheng and Xie, 60 cases were randomly divided into two groups and treated for three months: the treatment group received BBR (0.5 g, tid) in combination with glipizide (5 mg, bid) and metformin (0.5 g, tid), while the control group took glipizide (5 mg, bid) and metformin (0.5 g, tid) only; results showed that treatment group showed better efficacies in the levels of FBG, homeostasis model assessment of insulin resistance (HOMA-IR), TNF-*α*, IL-6, and CRP than those of control group (*P* < 0.05), indicating that BBR may be helpful to improve the disorders of glucose metabolism and insulin sensitivity [83]. Yin et al. observed the effect of using BBR combined with metformin on primary T2DM. 60 patients were assigned randomly to treatment group and control group; BBR (0.3 g, tid) plus metformin (0.5 g, tid) were administrated orally in the treatment group, while the subjects in the control group took metformin (0.5 g, tid) alone for 24 weeks. Indicators of HbA1C, FBG, 2hPG, fasting insulin (FINS), and lipid series (CHO, TG, HDL-C, and LDL-C) were monitored. Results showed that the levels of HbAlC, FBG, 2hBG, FINS, TG, HDL-C, and LDL-C all decreased in both groups, and there was significant difference between before and after the treatment (*P* < 0.05). The treatment group showed better effects on the above indicators than those of the control group, and statistical difference was notable (*P* < 0.05) [84]. Li et al. demonstrated the efficacy and safety of combined BBR and glipizide on patients with T2DM. 152 cases of diabetic patients from BBR group (51 cases), glipizide group (50 cases), and BBR combined with glipizide group (51 cases) were treated for 12 weeks. FPG and PBG were all decreased effectively in three groups, and the effective rate of BBR combined with glipizide was the highest (*P* < 0.05) when compared with BBR group and glipizide group. Glipizide had no effects on metabolism of blood lipids but had some effects on improving insulin resistance; administration of BBR or BBR combined with glipizide on diabetic patients could not only improve insulin resistance and recover the islet function but also effectively decrease the level of blood lipids, and no obvious adverse effects were observed in this research [85]. A systemic review [17] including 14 randomized trials indicated that the combined therapies of BBR and lifestyle modification had better effects on lowering the blood glucose and regulating the blood lipids, compared with lifestyle modification alone or plus placebo. The glucose-lowering efficacy of BBR was similar to those of oral hypoglycemic agents including metformin, glipizide, or rosiglitazone but showed better antidyslipidemic effect when compared with those of oral hypoglycemic agents. The combined therapies with BBR and the oral hypoglycemic agents showed a better glycemic control compared with oral hypoglycemic agents alone, but there were not significant improvements in TG, high-density lipoprotein cholesterol (HDL-C) and LDL-C, and a mild improvement in CHO, which need to be further demonstrated. The results suggested that BBR seems to be as effective as the conventional oral hypoglycemic agents, but most clinical trials included in this review were conducted in China, and quality assessment is low. Multicenter, well-controlled, and long-term clinical trials are still needed to provide the better evidence of BBR in the treatment of DM.

### 3.2. Applicable Stage and Syndrome

BBR is the main active constituent in* Rhizoma Coptidis* (*RC*), accounting for 6.88% to 13.64% [86], and it is consistent with* RC* in medicinal properties, flavor, and efficacy [87]. Wu and Wei [88] observed efficacy of BBR in treating T2DM. Seventy-two cases were assigned to obese group and nonobese group, and BBR (0.02 mg/kg) was administrated orally for 8 to 10 weeks. Results showed that insulin resistance and body mass index (BMI) of all cases improved after treatment, compared with before treatment (*P* < 0.01). BMI of obese group reduced more significantly than that of nonobese group (*P* < 0.01), indicating that BBR was more applicable to prediabetes and the early stage of T2DM characterized by insulin resistance and obesity. Tang and Shen [89] observed the hypoglycemic efficacy of BBR on different TCM syndromes of T2DM; one hundred and twenty patients were assigned to four groups, including syndromes of yin deficiency and excess heat, damp-heat encumbering in the spleen, dual deficiency of qi and yin, and blood stasis in collaterals. BBR (0.5 g) was orally given three times daily for three months. Results indicated that BBR had better hypoglycemic effects on syndrome of damp-heat encumbering in the spleen than other groups, and BMI decreased more significantly in group of damp-heat encumbering in the spleen (*P* < 0.05) than those of other groups (*P* > 0.05), which was consistent with properties of* RC*. The characteristics of splenic damp-heat syndrome commonly include bitter taste in the mouth, obesity, abdominal stuffiness and fullness, sticky fetid stool, yellow-greasy coating, and slippery and rapid pulse [90]. BBR could significantly improve the symptoms of splenic damp-heat syndrome of diabetic patients, as well as controlling the blood glucose effectively [91].

### 3.3. Reasonable Dose and Dose Formulation

Currently, BBR is recommended in Chinese medicine at doses of 0.2–1.0 g/day in dose forms such as tablets and capsules for treating various chronic diseases, especially for T2DM [45]. In a clinical research, Zhang et al. [82] confirmed that oral administration of BBR at such a dose of 1.0 g/day/person for 3 months is effective and safe in the treatment of T2DM and dyslipidemia. Guo and Zhao [92] observed that BBR showed obvious dose-effect relationship when used for improving the glucose tolerance. However, many researches that using BBR for T2DM overrun common dose, even up to 1.5 g/d [91]. Although there has been no report on the fact that increasing the dose of BBR causes hypoglycemia or other adverse effects, the report on the safety of using BBR with large doses and in the long term is lacking. Due to the poor bioavailability and solubility of BBR, it has been reported that the maximum concentration (*C*_max⁡_) of BBR in plasma was 0.4 ng/mL after a single oral dose of 400 mg of BBR in humans [93], and the therapeutic effect has been improved by making BBR hydrochloride liposome and cyclodextrin inclusion compound [20].

Due to the limitation of poor solubility and bioavailability, the form of compound or polyherbal formulations was produced. Fourteen BBR derivatives were synthesized and their antihyperglycemic effect was showed in alloxan-induced mice [94]. BBR derivatives, such as dihydroberberine (dhBBR) and 8,8-dimethyldihydroberberine (Di-MeBBR), have been demonstrated to possess better stability and bioavailability compared with BBR [95, 96]. In diet-induced obese (DIO) mice, Di-MeBBR decreased the adiposity, tissue triglyceride accumulation, and insulin resistance and improved glucose tolerance at a dose of 15 mg/kg. When administered to db/db mice with a dose of 50 mg/kg, Di-MeBBR effectively decreased FBG and TG, alleviated insulin resistance, and showed better efficacies than dhBBR [95, 96]. Nanoparticles mediated drug delivery systems containing phytochemicals from traditional medicines having advanced pharmacokinetic and pharmacodynamic properties may enhance the bioavailability of the drug [97]. The study by Torchilin VP suggested that O-hexadecyl-dextran entrapped berberine chloride nanoparticles (BC-HDD NPs), the nanotized form of BBR, were effective at 20-fold lower concentration than that of native BBR in preventing high glucose induced oxidative stress, mitochondrial depolarization. When applied to high glucose stressed hepatocytes, the enhanced effectiveness can be credited to not only its nanotized form but also its longer availability inside the cells as achieved by the designed and synthesized BC-HDD NPs [98]. Pund et al. described the development and characterization of self-nanoemulsifying drug delivery system (SNEDDS) of BBR with improved solubility, dissolution, and in vivo therapeutic efficacy. The in vitro rate and extent of release of BBR from SNEDDS were significantly higher than BBR alone. Chick chorioallantoic membrane assay revealed potent antiangiogenic activity of SNEDDS of BBR, which demonstrated that the SNEDDS of BBR was a promising strategy for improving its therapeutic efficacy and had potential application in the treatment of chronic inflammatory conditions, such as DM [99].

### 3.4. Toxicity and/or Side Effects

According to the regulations of Singapore government in 1976, BBR was forbidden to be used because it was deemed that a shortage of glucose 6 phosphate dehydrogenase (G6PD) could be caused after a pregnant woman or a newborn baby takes BBR, which may lead to hemolytic jaundice of the newborn baby [100]. Liao [101] carried out a study where he provided* RC* decoction to 22 newborn babies in hospital, among which three were short of G6PD. According to his observation, they concluded that the intake of* RC* within the dose range was safe and would not cause hemolytic jaundice or any other side effects for either newborn babies that were short of G6PD or normal ones. Ma et al. [102] identified that the median acute oral lethal dose of the total extract of* RC* was 2.95 g/kg in mice. The toxic constituents of* RC* were the alkaloids, mainly BBR. Generally, BBR in combination with other natural plants shows very less toxicity and side effects [20]. Kheir et al. [103] showed that the administrated method, as well as the dose of BBR, was significant factor that could affect acute toxicity of BBR. They provided the mice with different doses of BBR in different administrated ways and found that LD50 of intravenous injection and intraperitoneal injection were, respectively, 9.0386 g·kg^−1^ and 57.6103 g·kg^−1^ and that intragastric administration caused no LD50. Moreover, the measured concentrations of drug absorption were different under different ways of administration; the concentration under intragastric administration was the lowest. Na et al. [104] evaluated the effectiveness and safety of BBR in the treatment of T2DM, and the treated group that used BBR alone or BBR plus Western hypoglycemic drugs had no side effects of hypoglycemia, although mild gastrointestinal reactions existed, including diarrhea and constipation, and the adverse reactions of treated group had no significant difference compared with those of placebo and lifestyle intervention group (*P* > 0.05).

## 4. Summary

The rapidly increasing DM is becoming a predominant health problem, affecting 92.4 million persons in China. With increasing incidence of obesity, T2DM is likely to become even more prevalent in the future. It has a significant impact on the quality of life and the number of deaths as well as on the financial resources of public health care system. The treatment of T2DM and its complications mainly depends on Western hypoglycemic drugs and/or insulin, but more and more patients have been concerned about the potential toxicity and side effects, and they failed to delay the progression of diabetic complications, and sometimes the clinical efficacy is far from satisfactory. Due to positive views of patients towards complementary and alternative medicine (CAM) therapies and the increased availability of them, CAM therapies are increasingly and frequently used globally [105–107]; the commonly used therapies are traditional Chinese medicines, acupuncture, nutritional supplements and advice, spiritual healing, and relaxation techniques [107]. Natural plants, especially Chinese herbal medicines, have built up a characteristic medical system directed by traditional Chinese medical theory and provided rational means for various diseases including DM [13]. Berberine (BBR) is a classical component that is commonly used for treating T2DM, thus arousing strong interests in the mechanisms of its hypoglycemic activity. In this review, we provided scientific evidence about the mechanisms of its hypoglycemic activity, the randomized controlled trials (RCTs) on effectiveness and safety of BBR, the dose, and toxicity. A number of scientific studies have demonstrated the hypoglycemic effect of BBR on T2DM; for example, BBR could improve insulin sensitivity and promote insulin secretion; BBR regulates glucose and lipid metabolism in liver via modulating the PPARs protein expression; BBR reduces intestinal absorption of glucose; BBR possesses antioxidant activities aimed at diabetic complications [19–28]. In recent years, BBR has been demonstrated to treat T2DM possibly via modulating the composition of gut microbiota (enrichment of beneficial microbiota and inhibition of harmful microbiota) [48, 56, 57]. The review above showed that BBR could be effective in improving the glucolipid metabolism. The modern pharmacological researches on BBR are actually developing and numerous scientific evidences are provided and reported.

Chinese herbal medicines, instead of component, are often used by Chinese doctors; we introduced traditional Chinese medical knowledge to Western people and make them understand more the traditional Chinese medical theory. BBR, consistent with* RC*, possesses bitter flavor and cold property. In TCM theory, bitter flavor is in direct opposition to sweet flavor, so the bitter flavor is an excellent approach to counteract sweet flavor [108]. BBR was more applicable to prediabetes and the early stage of T2DM characterized by insulin resistance and obesity; BBR had better hypoglycemic effects on splenic damp-heat syndrome, which may be related to the significant improvement of the symptoms of splenic damp-heat syndrome, such as bitter taste in the mouth, obesity, abdominal stuffiness and fullness, sticky fetid stool, yellow-greasy coating, and slippery and rapid pulse. The common dose of BBR is 0.2–1.0 g/day. The form of compound and polyherbal formulations is also common. Some conclusions were based on clinical experience, or the grade of evidence was low; therefore, several limitations existed in this review. Well-designed, large-scale, and high-quality multicenter RCTs are needed to evaluate the effects of BBR for T2DM and its complications, and long-term outcomes are also needed to be evaluated, including the safety profile and the effects on organ damages (microalbuminuria, arteriosclerosis).

## Abbreviations

DM: Diabetes mellitus
IDF: International Diabetes Federation
T2DM: Type 2 diabetes mellitus
TCM: Traditional Chinese medicine
BBR: Berberine
RC: Rhizoma Coptidis
OHA: Oral hypoglycemic agents
DPN: Diabetic nephropathy
DN: Diabetic neuropathy
AMP: Adenosine monophosphate
AMPK: Adenosine monophosphate- (AMP-) activated protein kinase
GLP-1: Glucagon-like protein-1
T1DM: Type 1 diabetes mellitus
FBG: Fasting blood glucose
InsR: Insulin receptor
GLUT1: Glucose transporter type 1
RBP-4: Retinol binding protein-4
IRS-1: Insulin receptor substrate-1
GLUT4: Glucose transporter type 4
cAMP: Cyclic AMP
FoxO1: Forkhead transcription factor O1
SREBP1: Sterol regulatory element-binding protein 1c
ChREBP: Carbohydrate responsive element-binding protein
HNF-4*α*: Hepatic nuclear factor 4 alpha
FAS: Fatty acid synthase
PPARs: Peroxisome proliferator-activated receptors
LPS: Lipopolysaccharides
TLR4: Toll-like receptor 4
HFD: High-fat diet-fed
SCFA: Short-chain fatty acid
MDA: Malondialdehyde
SOD: Superoxide dismutase
GSH-Px: Glutathione peroxidase
GSH: Glutathione
BUN: Blood urea nitrogen
Scr: Serum creatinine
24 h-UAlb: 24 h urinary albumin
AR: Aldose reductase
TNF-*α*: Tumor necrosis factor-*α*
IL-1*β*: Interleukin-1*β*
IL-6: Interleukin-6
PGs: Prostaglandins
iNOS: Inducible nitric oxide synthase
MMP9: Matrix metalloprotease 9
MCP-1: Monocyte chemoattractant protein-1
CRP: C-reaction protein
COX-2: Cyclooxygenase-2
IRS-1: Insulin receptor substrate-1
NF-*κ*B: Nuclear factor-kappa B
CHO: Cholesterol
TG: Triglyceride
LDL-C: Low-density lipoprotein cholesterol
LDLR: Low-density lipoprotein receptor
MAPK: Mitogen-activated protein kinase
ERK: Extracellular signal-regulated kinase
JNK: C-Jun N-terminal kinase
HbA1C: Hemoglobin A1c
PBG: Postload plasma glucose
ALT: Alanine aminotransferase
*γ*-GT: *γ*-glutamate transpeptidase
HDL-C: High-density lipoprotein cholesterol
BMI: Body mass index
*C*_max⁡_: Maximum concentration
G6PD: Glucose 6 phosphate dehydrogenase
CAM: Complementary and alternative medicine
RCTs: Randomized controlled trials
HOMA-IR: Homeostasis model assessment of insulin resistance
FINS: Fasting insulin.

## References

[1] International Diabetes Federation (2013). *IDF Diabetes Altas Globally*.

[2] Wang Z. J., Wang J., Chan P. (2013). Treating type 2 diabetes mellitus with traditional Chinese and Indian medicinal herbs. *Evidence-Based Complementary and Alternative Medicine*.

[3] Xie W. D., Zhao Y. N., Zhang Y. O. (2011). Traditional Chinese medicines in treatment of patients with type 2 diabetes mellitus. *Evidence-Based Complementary and Alternative Medicine*.

[4] Zimmet P., Alberti K. G. M. M., Shaw J. (2001). Global and societal implications of the diabetes epidemic. *Nature*.

[5] Asante E. (2013). Interventions to promote treatment adherence in type 2 diabetes mellitus. *British Journal of Community Nursing*.

[6] Agarwal A. A., Jadhav P. R., Deshmukh Y. A. (2014). Prescribing pattern and efficacy of anti-diabetic drugs in maintaining optimal glycemic levels in diabetic patients. *Journal of Basic and Clinical Pharmacy*.

[7] Nagpal J., Bhartia A. (2006). Quality of diabetes care in the middle- and high-income group populace: the Delhi Diabetes Community (DEDICOM) survey. *Diabetes Care*.

[8] Valerón P. F., de Pablos-Velasco P. L. (2013). Limitations of insulin-dependent drugs in the treatment of type 2 diabetes mellitus. *Medicina Clínica*.

[9] Authority of the Board of the American Socitey of Health-System Pharamcists (2012). *AHFS Drug Information*.

[10] Hanefeld M., Ganz X., Nolte C. (2014). Hypoglycemia and cardiac arrhythmia in patients with diabetes mellitus type 2. *Herz*.

[11] Shi H. B., Cai Q. (2013). Summary on adverse reactions of antidiabetic drugs. *Zhong Guo Yao Wu Jing Jie*.

[12] El-Kaissi S., Sherbeeni S. (2011). Pharmacological management of type 2 diabetes mellitus: an update. *Current Diabetes Reviews*.

[13] Li W. L., Zheng H. C., Bukuru J., De Kimpe N. (2004). Natural medicines used in the traditional Chinese medical system for therapy of diabetes mellitus. *Journal of Ethnopharmacology*.

[14] Wang Q. (2003). The present situation of TCM treatment for diabetes and its researches. *Journal of Traditional Chinese Medicine*.

[15] Tzeng T.-F., Liou S.-S., Liu I.-M. (2013). The selected traditional chinese medicinal formulas for treating diabetic nephropathy: perspective of modern science. *Journal of Traditional and Complementary Medicine*.

[16] Prabhakar P. K., Doble M. (2011). Mechanism of action of natural products used in the treatment of diabetes mellitus. *Chinese Journal of Integrative Medicine*.

[17] Dong H., Wang N., Zhao L., Lu F. (2012). Berberine in the treatment of type 2 diabetes mellitus: a systemic review and meta-analysis. *Evidence-Based Complementary and Alternative Medicine*.

[18] Dong H., Zhao Y., Zhao L., Lu F. (2013). The effects of berberine on blood lipids: a systemic review and meta-analysis of randomized controlled trials. *Planta Medica*.

[19] Derosa G., Maffioli P., Cicero A. F. G. (2012). Berberine on metabolic and cardiovascular risk factors: an analysis from preclinical evidences to clinical trials. *Expert Opinion on Biological Therapy*.

[20] Singh I. P., Mahajan S. (2013). Berberine and its derivatives: a patent review (2009–2012). *Expert Opinion on Therapeutic Patents*.

[21] Vuddanda P. R., Chakraborty S., Singh S. (2010). Berberine: a potential phytochemical with multispectrum therapeutic activities. *Expert Opinion on Investigational Drugs*.

[22] Chen Q. M., Xie M. Z. (1986). Studies on the hypoglycemic effect of *Coptis chinensis* and berberine. *Acta Pharmaceutica Sinica*.

[23] Zhang Q., Xiao X., Feng K. (2011). Berberine moderates glucose and lipid metabolism through multipathway mechanism. *Evidence-Based Complementary and Alternative Medicine*.

[24] Yang J., Yin J., Gao H., Xu L., Wang Y., Li M. (2012). Berberine improves insulin sensitivity by inhibiting fat store and adjusting adipokines profile in human preadipocytes and metabolic syndrome patients. *Evidence-based Complementary and Alternative Medicine*.

[25] Chueh W.-H., Lin J.-Y. (2011). Berberine, an isoquinoline alkaloid in herbal plants, protects pancreatic islets and serum lipids in nonobese diabetic mice. *Journal of Agricultural and Food Chemistry*.

[26] Chueh W. H., Lin J. Y. (2012). Protective effect of berberine on serum glucose levels in non-obese diabetic mice. *International Immunopharmacology*.

[27] Kong W.-J., Zhang H., Song D.-Q. (2009). Berberine reduces insulin resistance through protein kinase C-dependent up-regulation of insulin receptor expression. *Metabolism: Clinical and Experimental*.

[28] Kim S. H., Shin E.-J., Kim E.-D., Bayaraa T., Frost S. C., Hyun C.-K. (2007). Berberine activates GLUT1-mediated glucose uptake in 3T3-L1 adipocytes. *Biological and Pharmaceutical Bulletin*.

[29] Ko B.-S., Choi S. B., Park S. K., Jang J. S., Kim Y. E., Park S. (2005). Insulin sensitizing and insulinotropic action of berberine from *Cortidis rhizoma*. *Biological and Pharmaceutical Bulletin*.

[30] Zhang W., Xu Y.-C., Guo F.-J., Meng Y., Li M.-L. (2008). Anti-diabetic effects of cinnamaldehyde and berberine and their impacts on retinol-binding protein 4 expression in rats with type 2 diabetes mellitus. *Chinese Medical Journal*.

[31] Chen C., Zhang Y., Huang C. (2010). Berberine inhibits PTP1B activity and mimics insulin action. *Biochemical and Biophysical Research Communications*.

[32] Lee Y. S., Kim W. S., Kim K. H. (2006). Berberine, a natural plant product, activates AMP-activated protein kinase with beneficial metabolic effects in diabetic and insulin-resistant states. *Diabetes*.

[33] Hwang J.-T., Kwon D. Y., Yoon S. H. (2009). AMP-activated protein kinase: a potential target for the diseases prevention by natural occurring polyphenols. *New Biotechnology*.

[34] Kahn B. B., Alquier T., Carling D., Hardie D. G. (2005). AMP-activated protein kinase: ancient energy gauge provides clues to modern understanding of metabolism. *Cell Metabolism*.

[35] Yin J., Gao Z., Liu D., Liu Z., Ye J. (2008). Berberine improves glucose metabolism through induction of glycolysis. *The American Journal of Physiology—Endocrinology and Metabolism*.

[36] Cheng Z., Pang T., Gu M. (2006). Berberine-stimulated glucose uptake in L6 myotubes involves both AMPK and p38 MAPK. *Biochimica et Biophysica Acta—General Subjects*.

[37] Yu Y., Liu L., Wang X., Liu X., Xie L., Wang G. (2010). Modulation of glucagon-like peptide-1 release by berberine: *in vivo* and *in vitro* studies. *Biochemical Pharmacology*.

[38] Lu S.-S., Yu Y.-L., Zhu H.-J. (2009). Berberine promotes glucagon-like peptide-1 (7–36) amide secretion in streptozotocin-induced diabetic rats. *Journal of Endocrinology*.

[39] Xia X., Yan J., Shen Y. (2011). Berberine improves glucose metabolism in diabetic rats by inhibition of hepatic gluconeogenesis. *PLoS ONE*.

[40] Wang Z.-Q., Lu F.-E., Leng S.-H. (2008). Facilitating effects of berberine on rat pancreatic islets through modulating hepatic nuclear factor 4 alpha expression and glucokinase activity. *World Journal of Gastroenterology*.

[41] Berger J., Moller D. E. (2002). The mechanisms of action of PPARs. *Annual Review of Medicine*.

[42] Zhou J. Y., Zhou S. W., Zhang K. B. (2008). Chronic effects of berberine on blood, liver glucolipid metabolism and liver PPARs expression in diabetic hyperlipidemic rats. *Biological and Pharmaceutical Bulletin*.

[43] Schoonjans K., Staels B., Auwerx J. (1996). The peroxisome proliferator activated receptors (PPARs) and their effects on lipid metabolism and adipocyte differentiation. *Biochimica et Biophysica Acta: Lipids and Lipid Metabolism*.

[44] Huang C., Zhang Y., Gong Z. (2006). Berberine inhibits 3T3-L1 adipocyte differentiation through the PPAR*γ* pathway. *Biochemical and Biophysical Research Communications*.

[45] Yin J., Zhang H., Ye J. (2008). Traditional Chinese medicine in treatment of metabolic syndrome. *Endocrine, Metabolic and Immune Disorders—Drug Targets*.

[46] Liu L., Yu Y.-L., Yang J.-S. (2010). Berberine suppresses intestinal disaccharidases with beneficial metabolic effects in diabetic states, evidences from in vivo and in vitro study. *Naunyn-Schmiedeberg's Archives of Pharmacology*.

[47] Liu L., Deng Y., Yu S., Lu S., Xie L., Liu X. (2008). Berberine attenuates intestinal disaccharidases in streptozotocin-induced diabetic rats. *Pharmazie*.

[48] Han J., Lin H., Huang W. (2011). Modulating gut microbiota as an anti-diabetic mechanism of berberine. *Medical Science Monitor*.

[49] Hotamisligil G. S. (2006). Inflammation and metabolic disorders. *Nature*.

[50] Cani P. D., Delzenne N. M. (2009). The role of the gut microbiota in energy metabolism and metabolic disease. *Current Pharmaceutical Design*.

[51] Li Z. X., Wang X. H., Zhao J. H. (2001). Studies on in vitro antibacterial activity of Coptis chinensis against 252 strains inclinic with muellerhinton agar. *Zhong Cao Yao*.

[52] Iwazaki R. S., Endo E. H., Ueda-Nakamura T., Nakamura C. V., Garcia L. B., Filho B. P. D. (2010). *In vitro* antifungal activity of the berberine and its synergism with fluconazole. *Antonie van Leeuwenhoek*.

[53] Yan D., Jin C., Xiao X.-H., Dong X.-P. (2008). Antimicrobial properties of berberines alkaloids in *Coptis chinensis* Franch by microcalorimetry. *Journal of Biochemical and Biophysical Methods*.

[54] Ley R. E., Turnbaugh P. J., Klein S., Gordon J. I. (2006). Microbial ecology: human gut microbes associated with obesity. *Nature*.

[55] DiBaise J. K., Zhang H., Crowell M. D., Krajmalnik-Brown R., Decker G. A., Rittmann B. E. (2008). Gut microbiota and its possible relationship with obesity. *Mayo Clinic Proceedings*.

[56] Xie W., Gu D., Li J., Cui K., Zhang Y. (2011). Effects and action mechanisms of berberine and *Rhizoma coptidis* on gut microbes and obesity in high-fat diet-fed C57BL/6J mice. *PLoS ONE*.

[57] Zhang X., Zhao Y., Zhang M. (2012). Structural changes of gut microbiota during berberine-mediated prevention of obesity and insulin resistance in high-fat diet-fed rats. *PLoS ONE*.

[58] Li Z., Geng Y. N., Jiang J. D., Kong W. J. (2014). Antioxidant and anti-inflammatory activities of berberine in the treatment of diabetes mellitus. *Evidence-Based Complementary and Alternative Medicine*.

[59] Xie W., Du L. (2011). Diabetes is an inflammatory disease: evidence from traditional Chinese medicines. *Diabetes, Obesity and Metabolism*.

[60] Singh J., Kakkar P. (2009). Antihyperglycemic and antioxidant effect of *Berberis aristata* root extract and its role in regulating carbohydrate metabolism in diabetic rats. *Journal of Ethnopharmacology*.

[61] Golbidi S., Laher I. (2010). Antioxidant therapy in human endocrine disorders. *Medical Science Monitor*.

[62] Zhou J.-Y., Zhou S.-W. (2011). Protective effect of berberine on antioxidant enzymes and positive transcription elongation factor b expression in diabetic rat liver. *Fitoterapia*.

[63] Bhutada P., Mundhada Y., Bansod K. (2011). Protection of cholinergic and antioxidant system contributes to the effect of berberine ameliorating memory dysfunction in rat model of streptozotocin-induced diabetes. *Behavioural Brain Research*.

[64] Wang Y., Campbell T., Perry B., Beaurepaire C., Qin L. (2011). Hypoglycemic and insulin-sensitizing effects of berberine in high-fat diet- and streptozotocin-induced diabetic rats. *Metabolism: Clinical and Experimental*.

[65] Liu W.-H., Hei Z.-Q., Nie H. (2008). Berberine ameliorates renal injury in streptopzotocin-induced diabetic rats by suppression of both oxidative stress and aldose reductase. *Chinese Medical Journal*.

[66] Lan T., Shen X., Liu P. (2010). Berberine ameliorates renal injury in diabetic C57BL/6 mice: involvement of suppression of SphK-S1P signaling pathway. *Archives of Biochemistry and Biophysics*.

[67] Liu W., Liu P., Tao S. (2008). Berberine inhibits aldose reductase and oxidative stress in rat mesangial cells cultured under high glucose. *Archives of Biochemistry and Biophysics*.

[68] Yang H. Z., Zhou M. M., Zhao A. H. (2009). Study on effects of baicalin, berberine and astragalus polysaccharides and their combinative effects on aldose reductase in vitro. *Zhong Yao Cai*.

[69] Dregan A., Charlton J., Chowienczyk P., Gulliford M. C. (2014). Chronic inflammatory disorders and risk of type 2 diabetes mellitus, coronary heart disease, and stroke: a population-based cohort study. *Circulation*.

[70] Jeong H. W., Hsu K. C., Ham M. (2009). Berberine suppresses pro-inflammatory responses through AMPK activation in macrophages. *American Journal of Physiology—Endocrinology and Metabolism*.

[71] Lou T., Zhang Z., Xi Z. (2011). Berberine inhibits inflammatory response and ameliorates insulin resistance in hepatocytes. *Inflammation*.

[72] Jiang Q., Liu P., Wu X. (2011). Berberine attenuates lipopolysaccharide-induced extracelluar matrix accumulation and inflammation in rat mesangial cells: involvement of NF-*κ*B signaling pathway. *Molecular and Cellular Endocrinology*.

[73] Wang Y. (2014). Attenuation of berberine on lipopolysaccharide-induced inflammatory and apoptosis responses in *β*-cells via TLR4-independent JNK/NF-*κ*B pathway. *Pharmaceutical Biology*.

[74] Tang L. Q., Wei W., Chen L. M., Liu S. (2006). Effects of berberine on diabetes induced by alloxan and a high-fat/high-cholesterol diet in rats. *Journal of Ethnopharmacology*.

[75] Kong W., Wei J., Abidi P. (2004). Berberine is a novel cholesterol-lowering drug working through a unique mechanism distinct from statins. *Nature Medicine*.

[76] Kong W. J., Liu J., Jiang J. D. (2006). Human low-density lipoprotein receptor gene and its regulation. *Journal of Molecular Medicine (Berlin)*.

[77] Abidi P., Zhou Y., Jiang J.-D., Liu J. (2005). Extracellular signal-regulated kinase-dependent stabilization of hepatic low-density lipoprotein receptor mRNA by herbal medicine berberine. *Arteriosclerosis, Thrombosis, and Vascular Biology*.

[78] Lee S, Lim H. J., Park J. H., Lee K. S., Jang Y., Park H. Y. (2007). Berberine-induced LDLR up-regulation involves JNK pathway. *Biochemical and Biophysical Research Communications*.

[79] Brusq J.-M., Ancellin N., Grondin P. (2006). Inhibition of lipid synthesis through activation of AMP kinase: an additional mechanism for the hypolipidemic effects of berberine. *Journal of Lipid Research*.

[80] Yin J., Xing H., Ye J. (2008). Efficacy of berberine in patients with type 2 diabetes mellitus. *Metabolism: Clinical and Experimental*.

[81] Zhang H., Wei J., Xue R. (2010). Berberine lowers blood glucose in type 2 diabetes mellitus Patients through increasing insulin receptor expression. *Metabolism: Clinical and Experimental*.

[82] Zhang Y., Li X., Zou D. (2008). Treatment of type 2 diabetes and dyslipidemia with the natural plant alkaloid berberine. *The Journal of Clinical Endocrinology & Metabolism*.

[83] Sheng Z. X., Xie D. H. (2010). Therapeutic effect of berberine on the levels of inflammatory factors in type 2 diabetic patients. *New Medicine*.

[84] Yin S. L., Yin H. Q., Li C. H., Liu C. M. (2011). The effects of berberine hydrochloride on blood glucose, insulin and lipids levels in newly diagnosed type 2 diabetic patients. *Xu Zhou Yi Xue Yuan Xue Bao*.

[85] Li Z., Liu L. H. (2007). Therapeutic efficacy of combined berberine and glipizide on type 2 diabetes mellitus. *Yi Xue Lin Chuang Za Zhi*.

[86] Pu S. B., Xu H. X., Zhang Y. H. (1999). Individual difference of growth condition and alkaloid content of Chinese gold thread (*Coptis chinensis*). *Zhong Cao Yao*.

[87] Zhang Y. P., Han L., Yang Q. H. (2012). Discussion on the medicinal properties and efficacy of berberine. *Zhong Guo Yi Yao Ke Xue*.

[88] Wu D., Wei J. (2009). Clinical observation on berberine treating type 2 diabetes mellitus. *Acta Universitatis Medicinalis Nanjing*.

[89] Tang H., Shen Y. (2008). Clinical study on hypoglycemic effect of berberine on different TCM syndromes of type 2 diabetes. *Shang Hai Zhong Yi Yao Za Zhi*.

[90] Guo J., Chen H. D., Song J., Wang J., Zhao L., Tong X. (2004). Syndrome differentiation of diabetes by the traditional Chinese medicine. *Evidence-Based Complementary and Alternative Medicine*.

[91] Zhang T., Chen M. J. (2009). Research progresses on the anti-hyperglycemic effect of berberine. *Zhong Guo Shi Yong Yi Yao*.

[92] Guo B., Zhao H. X. (2006). Hypoglycemic pharmacodynamics and clinical efficacy of berberine. *Zhong Guo Yao Ye*.

[93] Hua W., Ding L., Chen Y., Gong B., He J., Xu G. (2007). Determination of berberine in human plasma by liquid chromatography-electrospray ionization-mass spectrometry. *Journal of Pharmaceutical and Biomedical Analysis*.

[94] Bian X. L., He L. C., Yang G. D. (2006). Synthesis and anti-hyperglycemic evaluation of various protoberberine derivatives. *Bioorganic & Medicinal Chemistry Letters*.

[95] Cheng Z., Chen A. F., Wu F. (2010). 8,8-Dimethyldihydroberberine with improved bioavailability and oral efficacy on obese and diabetic mouse models. *Bioorganic & Medicinal Chemistry*.

[96] Turner N., Li J.-Y., Gosby A. (2008). Berberine and its more biologically available derivative, dihydroberberine, inhibit mitochondrial respiratory complex I: a mechanism for the action of berberine to activate amp-activated protein kinase and improve insulin action. *Diabetes*.

[97] Torchilin V. P. (2014). Multifunctional, stimuli-sensitive nanoparticulate systems for drug delivery. *Nature Reviews Drug Discovery*.

[98] Kapoor R., Singh S., Tripathi M., Bhatnagar P., Kakkar P., Gupta K. C. (2014). O-hexadecyl-dextran entrapped berberine nanoparticles abrogate high glucose stress induced apoptosis in primary rat hepatocytes. *PLoS ONE*.

[99] Pund S., Borade G., Rasve G. (2014). Improvement of anti-inflammatory and anti-angiogenic activity of berberine by novel rapid dissolving nanoemulsifying technique. *Phytomedicine*.

[100] Yang S. Y., Wang X. H. (2008). Discussion between Chinese herbal researches on skin infections of newborn infants and ‘jaundice of the newborn baby caused by *Rhizoma Coptidis*’. *Yi Xue Yan Jiu*.

[101] Liao C. L. (1982). *Preliminary Observation on Physiological Jaundice (Fetal Jaundice) of the Newborn Baby Related by Yinchen Hao Decoction and Rhizoma Coptidis*.

[102] Ma B. L., Ma Y. M., Shi R. (2010). Identification of the toxic constituents in Rhizoma Coptidis. *Journal of Ethnopharmacology*.

[103] Kheir M. M., Wang Y., Hua L. (2010). Acute toxicity of berberine and its correlation with the blood concentration in mice. *Food and Chemical Toxicology*.

[104] Na Q. M. G., Zhao T. Y., He M., Tian C. (2012). Effectiveness and safety of berberine in the treatment of type 2 diabetes: a systematic review. *Zhong Guo Xun Zheng Yi Xue Za Zhi*.

[105] Vincent C., Furnham A. (1996). Why do patients turn to complementary medicine? An empirical study. *British Journal of Clinical Psychology*.

[106] Bell R. A., Suerken C. K., Grzywacz J. G., Lang W., Quandt S. A., Arcury T. A. (2006). Complementary and alternative medicine use among adults with diabetes in the United States. *Alternative Therapies in Health and Medicine*.

[107] Lee M.-S., Lee M. S., Lim H.-J., Moon S.-R. (2004). Survey of the use of complementary and alternative medicine among Korean diabetes mellitus patients. *Pharmacoepidemiology and Drug Safety*.

[108] Tu X., Xie C. G., Wang F. (2013). *Fructus mume* formula in the treatment of type 2 diabetes mellitus: a randomized controlled pilot trial. *Evidence-Based Complementary and Alternative Medicine*.

